# Quality measurement on shear wave speed imaging: diagnostic value in differentiation of thyroid malignancy and the associated factors

**DOI:** 10.18632/oncotarget.13996

**Published:** 2016-12-16

**Authors:** Bo-Ji Liu, Chong-Ke Zhao, Hui-Xiong Xu, Yi-Feng Zhang, Jun-Mei Xu, Dan-Dan Li, Xiao-Wan Bo, Xiao-Long Li

**Affiliations:** ^1^ Department of Medical Ultrasound, Shanghai Tenth People's Hospital, Ultrasound Research and Education Institute, Tongji University School of Medicine, Shanghai 200072, China; ^2^ Thyroid Institute, Tongji University School of Medicine, Shanghai Center for Thyroid Diseases, Shanghai 200072, China

**Keywords:** shear wave elastography, shear wave speed imaging, thyroid nodule, quality measurement

## Abstract

To evaluate the associated factors for quality measurement (QM) on shear wave speed (SWS) imaging and the additional value of QM for differentiation of thyroid nodules. A consecutive series of 238 patients with 254 thyroid nodules were enrolled. They were all evaluated by conventional ultrasound and SWS imaging and were finally proven pathologically. QM was used to assess whether SWS propagation was authentic and was classified as high QM and Low QM. Twelve variables were analyzed to evaluate the associated factors for QM using binary logistic regression. Receiver operating characteristic (ROC) curve was plotted on SWS and SWS+QM. Sensitivity, specificity, positive predictive value (PPV), negative predictive value (NPV), accuracy and area under ROC curve (AUC) were calculated. The study included 170 benign thyroid nodules (160 high QM and 10 low QM) and 84 malignant thyroid nodules (56 high QM and 28 low QM) (*P* < 0.001). The mean SWS of benign and malignant nodules were 2.51 ± 0.47 m/s and 3.43 ± 1.21 m/s respectively (*P* < 0.001). The sensitivities, specificities, PPVs, NPVs, accuracies and AUCs were 77.4%, 80.0%, 65.7%, 87.7%, 79.1%, 0.82 for SWS alone with SWS ≥ 2.78 m/s; 33.3–34.5%, 91.2–94.1%, 65.9–73.7%, 73.8–74.1%, 72.4–74.0%, 0.63–0.64 for QM alone and 84.5–85.7%, 72.4–75.9%, 60.5–63.4%, 90.8–91.0%, 76.8–78.7%, 0.79–0.80 for SWS+QM. Nodule depth was identified to be the strongest associated factor for QM of SWS, followed by malignancy and SWS. In conclusion, QM for thyroid nodule is associated with nodule depth, malignancy, and SWS. QM improves the specificity in comparison with SWS alone, whereas SWS+QM does not improve the overall diagnostic performance.

## INTRODUCTION

Thyroid nodules are common in clinical practice [1, 2]. Most thyroid nodules are benign and only 7%–15% of them are malignant [1, 3]. High resolution ultrasound (US) is the most widely used examination method for thyroid nodules. The diagnosis of thyroid malignancy mainly depends on the features on US including solid component, hypoechogenicity or markedly hypoechogenicity, microcalcification, irregular margin, taller than wide shape, suspicious lateral lymph node, and so on [1, 4]. Based on the above mentioned US characteristics, the sensitivity and specificity vary in the range of 26%–87% and 53%–93% respectively.

US elastography is a complement to conventional US, which provides stiffness information of thyroid lesions [5–12]. There are two kinds of US elastography: strain elastography (SE) and shear wave elastography (SWE) [13]. SE is a qualitative method which depends on the tissue stiffness and deformation. SWE is a quantitative measurement, of which the transverse shear wave propagation is generated when the target tissue is excited by push pulse transmitted from the transducer. It is evaluated as shear wave speed (SWS, m/s) or shear modulus (KPa). The diagnostic performance is variable that the sensitivity of SE ranges from 57% to 92% [14–17] and the sensitivity of SWE ranges from 56% to 97% [18–21].

For previous SWE techniques, no quality measurement (QM) is available to evaluate whether SWS propagation is reliable or adequate. In fact, invalid SWS measurement may occur on the condition of cystic portions, macrocalcifications, thyroid malignancy, unstable elastography images, patient or transducer motion, which causes confusion when interpreting the SWE images. QM for SWS imaging might be a solution for this dilemma, which would help the operators to understand where the SWS measurement within the lesion is accurate and where the shear wave region of interest (SW-ROI) placement should be avoided. Two-dimensional (2D) quality map is developed to display the SW quality using different colors in a recent SWS imaging technique (i.e. virtual touch tissue imaging quantification, VTIQ; Siemens Medical Solutions, Mountain View, CA, USA), in which the green color represents high quality for SWS measurement while yellow or red color indicates low quality. Barr et al. [22] found that the sensitivity of SWS imaging for breast lesions increased remarkably from 50% to 93% by adding QM for analysis, while specificity had no statistically significant difference (94% to 89%). They believed that low QM might be a feature of breast malignancy.

Until present, no studies have been carried out to study the effect of QM to diagnosis of thyroid nodules. We hypothesized that QM for thyroid nodules might also improve the diagnostic value of SWS imaging. Therefore, the purpose of this study was to evaluate the associated factors for QM on SWS imaging and the additional value of QM for differentiation of thyroid nodules in comparison with SWS imaging alone.

## RESULTS

### Patients and nodules

**Figure 1:**
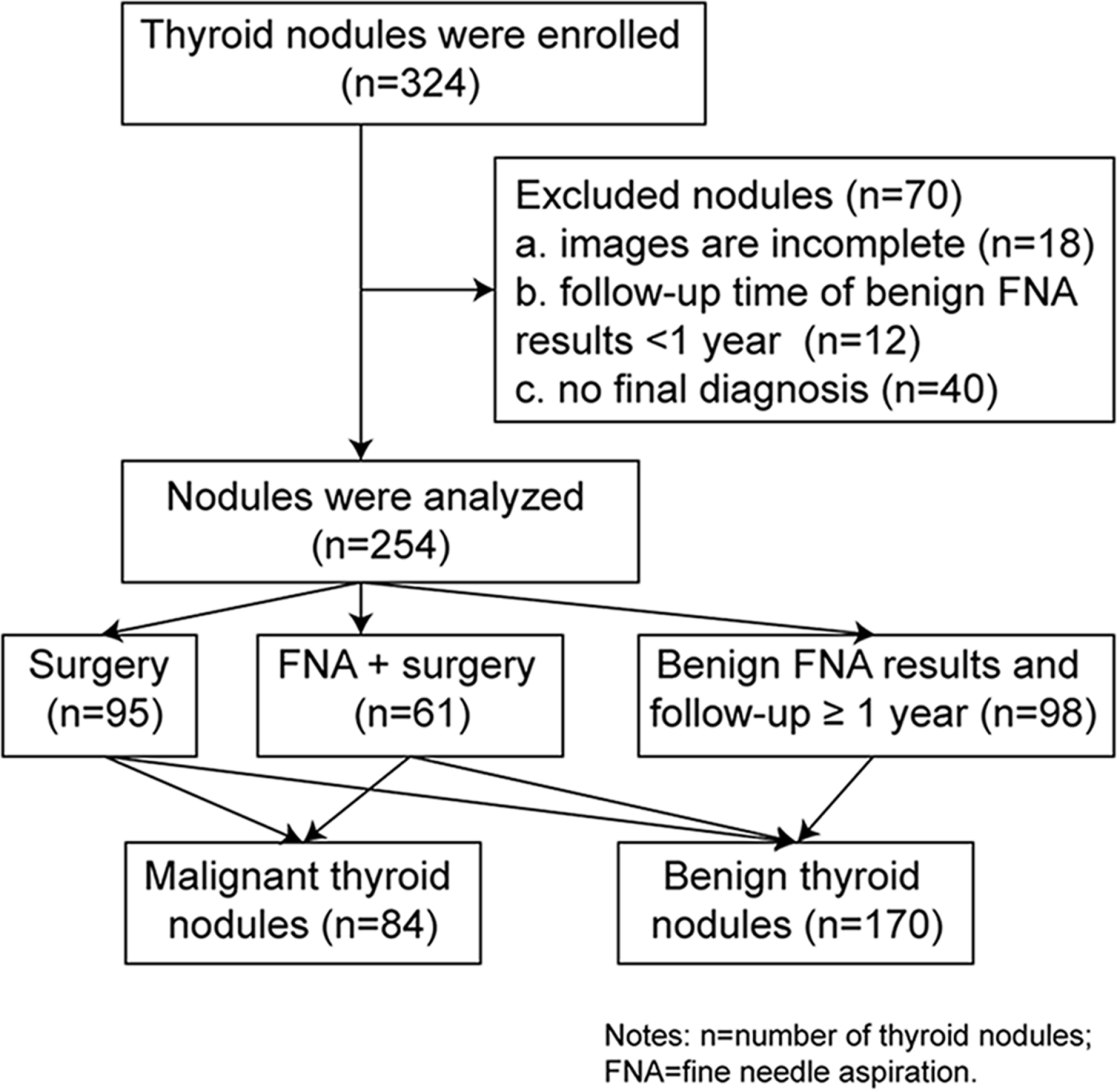
The flowchart of thyroid nodule selection

254 thyroid nodules (170 benign nodules and 84 malignant nodules, mean size: 12.9 ± 8.4 mm, range: 5–47 mm) from 238 patients (54 males and 184 females, mean age: 50.9 ± 11.9 years, range: 18–76 years) were enrolled for analysis in the study. The flowchart of nodule selection is shown in Figure 1. In the included 238 patients, 14 patients had 2 nodules and one patient had 3 nodules.

### Pathological and FNA cytological diagnosis

There were 254 thyroid nodules including 170 benign nodules (98 with benign FNA cytological results with follow-up for more than 1 year; 51 nodular goiters, 13 Hashimoto nodules, 6 adenomas, and 2 subacute thyroiditis, which were confirmed by pathology after surgery) and 84 malignant ones (79 papillary thyroid carcinomas, 2 follicular carcinomas, 2 medullary carcinomas and 1 undifferentiated carcinoma). All the malignancies were confirmed by pathological examination. Detailed pathological and FNA cytological results are presented in Table 1.

**Table 1:** Final diagnoses of thyroid nodules

	FNA cytological results (*n* = 159)	Pathological results (*n* = 156)	
		Benign (*n* = 72)	Malignant (*n* = 84)
FNA + follow up (*n* = 98)	Bethesda II (*n* = 98)	/	/
Surgery (*n* = 95)	/	31 nodular goiters, 8 hashimoto nodules, 4 adenomas, 2 subacute thyroiditis	46 papillary thyroid carcinomas, 2 follicular carcinomas, 1 medullary carcinomas, 1 undifferentiated carcinoma
FNA + surgery (*n* = 61)	Bethesda II (*n* = 20)	16 nodular goiters, 4 hashimoto nodules	*n* = 0
	Bethesda III (*n* = 14)	4 nodular goiters, 1 hashimoto nodules	9 papillary thyroid carcinomas
	Bethesda IV (*n* = 2)	2 adenomas	*n* = 0
	Bethesda V (*n* = 16)	*n* = 0	15 papillary thyroid carcinomas, 1 medullary carcinoma
	Bethesda VI (*n* = 9)	*n* = 0	9 papillary thyroid carcinomas

Abbreviations: FNA, fine needle aspiration; Bethesda II, benign; Bethesda III, atypia of undetermined significance/follicular lesion of undetermined significance; Bethesda IV, follicular neoplasm/suspicious for follicular neoplasm; Bethesda V, suspicious for malignancy; Bethesda VI, malignant.

### US and SWS imaging features

The basic characteristics, US and SWS imaging features of benign and malignant thyroid nodules are presented in Table 2. No statistically significant differences in gender, age and internal blood flow on color Doppler US were found between benign and malignant thyroid nodules (all *P* > 0.05). Small nodule size, hypoechogenicity, poorly defined margin, irregular configuration, taller than wide shape and microcalcification were more commonly found in thyroid malignancies than in benign nodules (all *P* < 0.05).

**Table 2:** Conventional US and SWS imaging features of benign and malignant throid nodules

Parameters	Benign	Malignant	Overall	*P*
Patients	159	79	238	/
Gender (male/female)	35/124	19/60	54/184	0.724
Mean age (years)	51.6 ± 11.0	49.6 ± 13.6	50.9 ± 11.9	0.215
Nodules	170	84	254	/
Mean size (mm)	13.7 ± 8.2	11.1 ± 8.7	12.9 ± 8.4	0.021*
Mean depth (mm)	15.6 ± 4.3	15.4 ± 3.7	15.5 ± 4.1	0.755
Echogenicity				< 0.001*
Hyperechoic (%)	2 (1.2)	0 (0.0)	2 (0.8)	
Isoechoic (%)	51 (30.0)	4 (4.8)	55 (21.7)	
Hypoechoic (%)	80 (47.1)	75 (89.3)	155 (61.0)	
Mixed (%)	37 (21.8)	5 (6.0)	42 (16.5)	
Margin				< 0.001*
Well defined (%)	127 (74.7)	41 (48.8)	168 (66.1)	
Poor defined (%)	43 (25.3)	43 (51.2)	86 (33.9)	
Shape				0.001*
Regular (%)	148 (87.1)	49 (58.3)	197 (77.6)	
Irregular (%)	22 (12.9)	35 (41.7)	57 (22.4)	
Height and width				< 0.001*
Height < width (%)	161 (94.7)	46 (54.8)	207 (81.5)	
Height > width (%)	9 (5.3)	38 (45.2)	47 (18.5)	
Calcifications				< 0.001*
No calcifications	128 (75.3)	35 (41.7)	163 (64.2)	
Microcalifications	42 (24.7)	49 (58.3)	91 (35.8)	
Vascularity				0.267
No internal flow	44 (25.9)	24 (28.6)	68 (26.8)	
Rare internal flow	80 (47.1)	44 (52.4)	124 (48.8)	
Rich internal flow	46 (27.1)	16 (19.0)	62 (24.4)	
Quality measurement (%)				
Reader 1:High QM	155 (91.2)	55 (65.5)	210 (82.7)	< 0.001*
Low QM	15 (8.8)	29 (34.5)	44 (17.3)	
Reader 2:High QM	160 (94.1)	56 (66.7)	216 (85.0)	< 0.001*
Low QM	10 (5.9)	28 (33.3)	38 (15.0)	
SWS (m/s)	2.51 ± 0.47	3.43 ± 1.21	2.82 ± 0.90	< 0.001*

Data are means ± standard deviations.

*Statistically significant difference.

Abbreviations: US, ultrasound; SWS, shear wave speed; QM, quality measurement.

**Figure 2:**
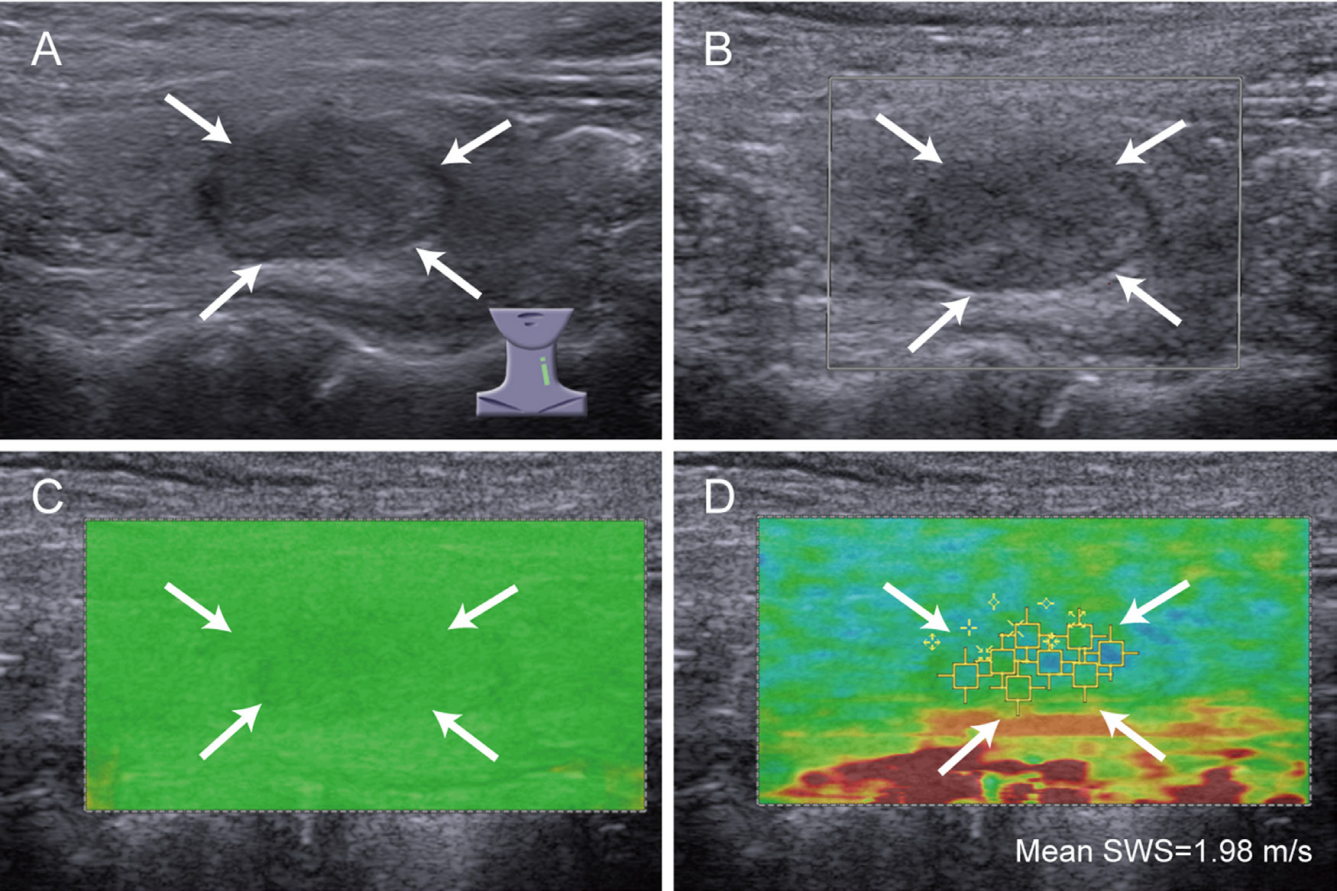
Images of high QM in benign thyroid nodule A 54-year-old woman has nodular goiter. (**A**) conventional ultrasound shows a 14 mm thyroid nodule (arrows) in left thyroid lobe, which is solid, hypoechoic and regular; (**B**) color Doppler ultrasound shows no color blood flow signal in the nodule (arrows); (**C**) SW-quality map shows almost green in the nodule (arrows), indicating high QM; (**D**) the mean SWS of the nodule (arrows) is 1.98 m/s on SW-velocity map.

**Figure 3:**
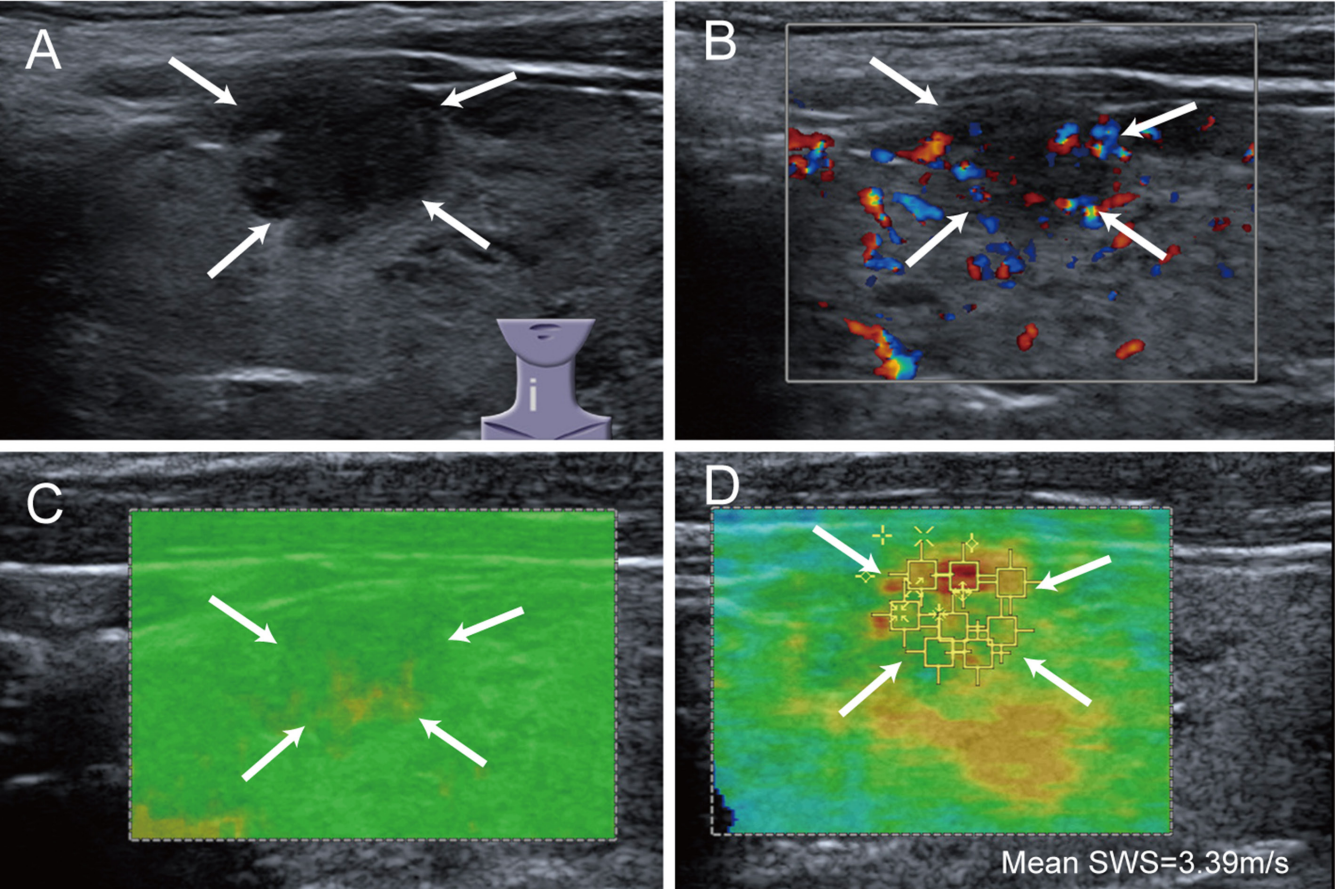
Images of low QM in malignant thyroid nodule A 39-year-old woman has papillary thyroid carcinoma. (**A**) conventional ultrasound shows a 12 mm thyroid nodule (arrows) in right thyroid lobe, which is solid, markedly hypoechoic and irregular; (**B**) color Doppler ultrasound shows sporadic color flow signals in the nodule (arrows); (**C**) SW-quality map shows that internal yellow portions are more than 20 percent of the nodule (arrows), indicating low QM; (**D**) the mean SWS of the nodule (arrows) is 3.39 m/s.

The SWS of malignant nodules (mean, 3.43 ± 1.21 m/s; range, 1.40–8.55 m/s) was statistically significant higher than that of benign nodules (mean, 2.51 ± 0.47 m/s; range, 1.52–4.54 m/s) (*P* < 0.001). Low QM was more often found in depth >15mm nodules (30/125, 24.0%) than depth ≤ 15 mm nodules (8/129, 6.2%) (*P* < 0.001). For reader 1, 155 benign nodules and 55 malignant nodules were allocated to high QM while 15 benign nodules and 29 malignant nodules were low QM; for reader 2, 160 benign nodules and 56 malignant nodules were allocated to high QM while 10 benign nodules and 28 malignant nodules were low QM. Low QM was more often happened in thyroid malignancy for both readers (both *P* < 0.001) (Figure 2, Figure 3). The kappa value for QM of two independent readers was 0.826.

### Diagnostic performances of SWS, QM, and SWS+QM

The diagnostic parameters of SWS, QM and SWS+QM are presented in Table 3. The sensitivity increased from 77.4% to 84.5% while specificity decreased from 80.0% to 75.9% by adding QM to SWS with no statistically significant difference (*P* = 0.238 for comparison of sensitivity and *P* = 0.433 for specificity). No difference for AUC was found between SWS and SWS+QM (0.82 vs 0.80, *P* = 0.517). In addition, no statistically significant difference for sensitivity, specificity, PPV, NPV, accuracy of SWS, QM and SWS+QM between two readers were found (all *P* > 0.05). For QM alone, although the specificity increased from 80% to 91.2–94.1% in comparison with SWS alone, the sensitivity decreased from 77.4% to 33.3–34.5% and the AUC decreased from 0.82 to 0.63–0.64 (all *P* < 0.05).

**Table 3:** The diagnostic performance of SWS and QM for differentiation of thyroid nodules

	Sensitivity (%)	Specificity (%)	PPV (%)	NPV (%)	Accuracy (%)	AUC (95% CI)
**All nodules (*n* = 254)**						
SWS ≥ 2.78 m/s	77.4 (65/84)	80.0 (136/170)	65.7 (65/99)	87.7 (136/155)	79.1 (201/254)	0.82 (0.76–0.88)
QM (reader 1)	34.5 (29/84)	91.2 (155/170)	65.9 (29/44)	73.8 (155/210)	72.4 (184/254)	0.63 (0.55–0.71)
QM (reader 2)	33.3 (28/84)	94.1 (160/170)	73.7 (28/38)	74.1 (160/216)	74.0 (188/254)	0.64 (0.56–0.71)
SWS + QM (reader 1)	85.7 (72/84)	72.4 (123/170)	60.5 (72/119)	91.1 (123/135)	76.8 (195/254)	0.79 (0.73–0.85)
SWS + QM (reader 2)	84.5 (71/84)	75.9 (129/170)	63.4 (71/112)	90.8 (129/142)	78.7 (200/254)	0.80 (0.74–0.86)
**< 10 mm nodules (*n*= 115)**						
SWS ≥ 2.76 m/s	66.7 (34/51)	84.4 (54/64)	77.3 (34/44)	76.1 (54/71)	76.5 (88/115)	0.77 (0.68–0.86)
QM (reader 1)	27.5 (14/51)	92.2 (59/64)	73.7 (14/19)	61.5 (59/96)	63.5 (73/115)	0.60 (0.49–0.70)
QM (reader 2)	25.5 (13/51)	95.3 (61/64)	81.3 (13/16)	61.6 (61/99)	64.3 (74/115)	0.60 (0.50–0.71)
SWS + QM (reader 1)	80.4 (41/51)	76.6 (49/64)	73.2 (41/56)	83.1 (49/59)	78.3 (90/115)	0.79 (0.70–0.87)
SWS + QM (reader 2)	78.4 (40/51)	79.7 (51/64)	75.5 (40/53)	82.3 (51/62)	79.1 (91/115)	0.79 (0.70–0.88)
**≥ 10 mm nodules (*n*= 139)**						
SWS ≥ 2.91 m/s	87.9 (29/33)	84.9 (90/106)	64.4 (29/45)	95.7 (90/94)	85.6 (119/139)	0.90 (0.84–0.96)
QM (reader 1)	45.5 (15/33)	90.6 (96/106)	60.0 (15/25)	84.2 (96/114)	79.9 (111/139)	0.68 (0.57–0.80)
QM (reader 2)	45.5 (15/33)	93.4 (99/106)	68.2 (15/22)	84.6 (99/117)	82.0 (114/139)	0.69 (0.58–0.80)
SWS + QM (reader 1)	90.9 (30/33)	77.4 (82/106)	55.6 (30/54)	96.5 (82/85)	80.6 (112/139)	0.84 (0.77–0.92)
SWS + QM (reader 2)	90.9 (30/33)	80.2 (85/106)	58.8 (30/51)	96.6 (85/88)	82.7 (115/139)	0.86 (0.78–0.93)
**Depth ≤ 15mm (*n* = 129)**						
SWS ≥ 2.91 m/s	80.0 (36/45)	79.8 (67/84)	67.9 (36/53)	88.2 (67/76)	79.8 (103/129)	0.82 (0.74–0.90)
QM (reader 1)	15.6 (7/45)	98.8 (83/84)	87.5 (7/8)	68.6 (83/121)	69.8 (90/129)	0.57 (0.46–0.68)
QM (reader 2)	15.6 (7/45)	98.8 (83/84)	87.5 (7/8)	68.6 (83/121)	69.8 (90/129)	0.57 (0.46–0.68)
SWS + QM (reader 1)	82.2 (37/45)	79.8 (67/84)	68.5 (37/54)	89.3 (67/75)	80.6 (104/129)	0.81 (0.73–0.89)
SWS + QM (reader 2)	82.2 (37/45)	79.8 (67/84)	68.5 (37/54)	89.3 (67/75)	80.6 (104/129)	0.81 (0.73–0.89)
**Depth > 15mm (*n* = 125)**						
SWS ≥ 2.76 m/s	74.4 (29/39)	88.4 (76/86)	74.4 (29/39)	88.4 (76/86)	84.0 (105/125)	0.82 (0.72–0.91)
QM (reader 1)	56.4 (22/39)	83.7 (72/86)	61.1 (22/36)	80.9 (72/89)	75.2 (94/125)	0.70 (0.60–0.81)
QM (reader 2)	53.8 (21/39)	89.5 (77/86)	70.0 (21/30)	81.1 (77/95)	78.4 (98/125)	0.72 (0.61–0.82)
SWS + QM (reader 1)	89.7 (35/39)	73.3 (63/86)	60.3 (35/58)	94.0 (63/67)	78.4 (98/125)	0.82 (0.74–0.90)
SWS + QM (reader 2)	87.2 (34/39)	80.2 (69/86)	66.7 (34/51)	93.2 (69/74)	82.4 (103/125)	0.84 (0.76–0.92)

Abbreviations: SWS, shear wave speed; QM, quality measurement; PPV, positive predictive value; NPV, negative predictive value; AUC, area under ROC curve; CI, confidence interval.

Subgroup analysis of diameter (< 10 mm group and ≥ 10 mm group) and depth (≤ 15 mm group and > 15 mm group) were performed. The sensitivity (87.9% vs 66.7%, *P* = 0.039) and AUC (0.90 vs 0.77, *P* = 0.019) of subgroup ≥ 10 mm were statistically higher than those of subgroup < 10 mm. It had no statistically significant differences for comparison of sensitivity, specificity and AUC between SWS and SWS+QM in both subgroups of diameter and depth (all *P* > 0.05). 6 malignant thyroid nodules in subgroup of < 10 mm and 1 malignant one in subgroup of ≥ 10 mm were correctly diagnosed after adding QM for diagnosis. No statistically significant difference was found on AUCs for QM in subgroup of diameter (*P* > 0.05), while it was present in subgroup of depth (*P* = 0.022 for reader 1 and *P* = 0.002 for reader 1). No statistically significant difference was found for sensitivity, specificity, PPV, NPV, accuracy of SWS, QM and SWS+QM between the two readers in two subgroups (all *P* > 0.05).

### Multivariate logistic regression analysis of QM

216 nodules showed high QM and 38 nodules low QM. Possible associated factors were subject to analysis including gender, age, malignancy, size, echogenicity, depth, margin, shape, height and width, calcification, vascularity, and SWS. Nodule depth (OR = 65.70, 95% CI: 10.43–413.91), malignancy (OR = 6.26, 95% CI: 1.54–23.36) and SWS (OR = 2.42, 95% CI: 1.09–5.41) were identified to be independent associated factors for low QM. Low QM was associated with nodule depth (OR = 165.05, 95% CI: 5.97–7095.32) and taller than wide shape (OR = 23.75, 95% CI: 1.55–363.24) in subgroup of < 10 mm whereas nodule depth (OR=31.13, 95% CI: 2.69–361.04) and SWS (OR = 7.06, 95% CI: 1.14–43.57) were identified to be independent associated factors in subgroup of ≥ 10 mm (Table 4).

**Table 4:** The independent associated factors for QM on SWS imaging for thyroid nodules

	OR	95% CI	*P*
All nodules (*n* = 254)			
Malignancy	6.26	1.54–23.36	0.010
Depth	65.70	10.43–413.91	< 0.001*
SWS	2.42	1.09–5.41	0.031
< 10 mm nodules (*n* = 115)			
Taller than wide shape	23.75	1.55–363.24	0.023
Depth	165.05	5.97–7095.32	0.014
≥ 10 mm nodules (*n* = 139)			
Depth	31.13	2.69–361.04	0.006

Abbreviations: QM, quality measurement; SWS, shear wave speed; OR, odds ratio; CI, confidence interval.

## DISCUSSION

Many studies have proven SWE with SWS measurement was useful for the differentiation of thyroid nodules [6–8, 18–22]. In the current study, the sensitivity and specificity of SWS imaging was 77.4% and 80.0%, which were consistent with the results of point SWS measurement using VTQ technique by Xu et al. [21] (i.e. sensitivity of 71.6% and specificity of 83.4%) and Zhang et al [18] (i.e. sensitivity of 75.0% and specificity of 82.2%). They were also consistent with the results of SWS imaging using Supersonic Shear wave Imaging (SSI) by Azizi et al. [23] (i.e. sensitivity of 79.3% and specificity of 71.5%). Therefore, although SWE has been reported to have a varied sensitivity in previous studies [18–21], SWE shows a similar diagnostic performance for thyroid nodules in recent studies [18–21], despite of different SWE techniques used.

It has been noted that QM of SW propagation in the target area is an important issue, which determines whether the SWS measurement is reliable or not. During the imaging process of SWE, measurement errors about the SW propagation may occur in the course of data acquisition and data processing. In the process of data acquisition, SWS imaging may be influenced by transducer compression, patient breath, carotid pulsation, and so on. On the other hand, low signal-to-noise ratio (SNR) may occur in the course of data processing. Even though excluding the factors of patient or transducer motion, SWS imaging was not applicable for all thyroid nodules. For instance, shear wave cannot propagate in cystic tissue, which always shows a non-color coded area on SWS imaging. In this study, all the nodules were solid or predominantly solid, thus this factor would not contribute to low QM.

Multivariate logistic regression analysis showed that the independent associated factors for low QM of overall thyroid nodules were nodule depth (OR: 65.7), malignancy (OR: 6.26) and nodule size (OR: 2.42). Nodule depth was identified to be the strongest factor of low QM and the underlying mechanism is that ARFI push pulse attenuates along with nodule depth. The SW induced by ARFI push becomes weaker with depth. Many studies have confirmed that lesion depth is related with the distance from the probe to the lesion [24, 25]. In the current study, low QM was more frequently found in deeper nodules than shallower nodules. This effect was more common for nodules ≤ 10 mm in diameter, as compared with those > 10 mm (OR: 165.05 vs. 31.13).

Malignancy was also identified as an independent associated factor for low QM (OR: 6.26) that 3.5% (7/170) benign nodules and 33.3% (28/84) malignant nodules showed low QM (*P* < 0.001). Therefore, low QM was more commonly found in thyroid malignancy. Tissue in homogeneity might be the cause for low QM in malignancy. Pathologically, thyroid malignancy has a mixed pathologic structure with solid cells, fibrosis, and adipose tissue; whereas benign thyroid nodule has a more uniform pathological structure. Tissue inhomogeneity in thyroid malignancy may interfere or block the transverse propagation of SW in the targeted tissue. In addition, when the SW propagation is interrupted by a sudden decrease or a sudden increase of tissue stiffness due to tissue inhomogeneity, the SW would attenuate significantly and thus lead to a low QM.

Interestingly, SWS was firstly revealed to be the independent associated factor for QM with high SWS associated with low QM. The underlying mechnism was unknown. One of the possible explanations is that high SWS is associated with high tissue stiffness, however, high stiffness tissue might be more inhomogeneous. As mentioned above, if there was a sudden decrease or a sudden increase of tissue stiffness, low MI would be encountered.

Although thyroid malignancy was associated with low QM, no substantial improvement in diagnostic performance was found when adding QM to SWS (AUC: 0.82 vs 0.81). The sensitivity increased slightly (from 77.4% to 84.5%) whereas the specificity (from 80.0% to 77.1%) decreased slightly and there were no statistically significant differences. Therefore, at the current stage, QM alone although increased the specificity significantly, it is not a valid method for differential diagnosis of thyroid nodules. It is more suitable to be used a reference to place SW-ROI at an appropriate area in the nodule.

The study had several limitations. First, it was a single-center study and sample size was not large enough, a multi-center study with larger sample size was expected in the future. Second, no previous studies about QM on thyroid lesions were published, thus, the definition of high and low QM need validation from more studies. Thirdly, the retrospective nature of the study could not avoid selection bias and future prospective study is needed. Finally, only one type of SWS imaging equipment was used in the study, whether the results were applicable for other elastography equipments need further clarification.

## MATERIALS AND METHODS

### Patients

The retrospective study was approved by the ethics committee of the university hospital and informal consent was waived due to the retrospective nature of the study. The reasons why the patients underwent US examination mainly included: thyroid nodules found by palpation; with compression symptom or discomfort in the cervical region; thyroid nodules on follow-up; thyroid nodules found in primary clinic. Inclusion criteria were as follows: (a) age of 18–80 years; (b) thyroid nodules with diameter ≥ 5 mm according to the recent guideline [26]; (c) patients underwent surgery or fine needle aspiration (FNA) after conventional US and SWS imaging examination; (d) without invasive procedures included fine needle aspiration (FNA) and thermal ablation before the examination; (e) solid or predominantly solid nodules (i.e. cystic portion < 25%); (g) without macrocalcifications (diameter of calcification > 1.0 mm) and posterior shadowing. Exclusion criteria included: (a) image data were incomplete (*n* = 18); (b) patients with benign FNA cytological results were lost to follow-up or follow-up periods were less than 12 months (*n* = 12); (c) patients with non-diagnostic, atypical or suspicious FNA cytological results and without confirmation from surgery (*n* = 40). Finally, 254 thyroid nodules (170 benign nodules and 84 malignant nodules, mean size: 12.9 ± 8.4 mm) from 238 patients (54 males and 184 females, mean age: 50.9 ± 11.9 years) were enrolled for analysis in the study from August 2014 to June 2015 (Figure 1).

### Conventional US and SWS imaging examination

All the thyroid nodules were subject to conventional US and SWS imaging examinations using S3000 US scanner (Siemens Medical Solutions, Mountain View, CA, USA). One of two board certified radiologists with 3 years’ experience with thyroid US and 2 years’ experience with thyroid SWS imaging completed all the US and SWE examinations. On conventional US, the 18L6 linear array transducer (Frequency range: 5.5–18MHz) was usually used to scan the thyroid and the nodule firstly, and on occasions the 9L4 linear array transducer (Frequency: 4–9MHz) was used when the nodule was too large or too deep. For SWS imaging, only the 9L4 linear array transducer was applied since the SWS imaging mode was not available with the 18L6 linear array transducer. A standard US examination including B-mode and color Doppler imaging was then performed. Patients were required to stretch out on the check-bed and keep their necks slightly extended. Neck skin was exposed enough. The target nodule was placed in the center of the US screen and the image was optimized. Transverse and longitudinal US images were obtained and saved for each target nodule, including color Doppler flow images.

SWS imaging was then performed with compression as slight as possible. Images of SWS imaging were obtained after patients holding breath for 5 seconds in the longitudinal plane of the target nodule after adjusting the size of sampling box. The sampling box was adjusted to cover the target thyroid nodules and part of the surrounding thyroid tissue. SW-quality mode was firstly initialed and a 2D color map of SW quality was displayed, of which the scale of the color map was fixed. The high quality is displayed in green, whereas poor quality in yellow or red. Afterwards, the SW-velocity mode was started and a 2D color map of SWS distribution was obtained. The color of the 2D SW-velocity map represents the SWS from high (red), intermediate (yellow or green), to low (blue). The thyroid nodule was highlighted by adjustment of velocity scale. The scale of SWS ranges from 0.5 to 10 m/s and adjustments of SW-velocity scale would not change the results of SWS measurement. The SW-ROIs (minimal size, 1.5 × 1.5 mm) were placed on the carefully selected areas on 2D SW-velocity map, which corresponded high quality areas on SW-quality map whereas poor quality areas were avoided. When the quality map is nearly red or yellow (low quality), we would try to find some small green areas on the quality map and put the ROI in these areas. And if none, the ROI would be put on the yellow areas since the quality decreases from green to yellow to red, and red indicates the lowest quality. At least seven SWS measurements were carried out for each lesion. The SW-ROI placement on the SW-velocity map was random for nodules with homogeneous SWS distribution. For those with heterogeneous SWS distribution, two SW-ROIs were placed on the highest SWS area and the lowest SWS area respectively, and the remaining five SW-ROIs were placed randomly, depending on the different colors visualized on SW-velocity map. All the images of conventional US and SWS imaging examinations were saved in the hard disk incorporated in the US system and were retrieved digitally for further analysis. QM of SWS added no more than 5 minutes and added no cost in this series.

### Image interpretation

For conventional US images, the target nodule was evaluated for nodule size (measured in longitudinal plane), echogenicity (hyperechoic, isoechoic, hypoechoic and mixedechoic), margin (well defined and poorly defined), shape (regular and irregular), height and width (wider than tall and taller than wide), calcifications (no calcifications; microcalcifications, less than 1.5 mm in diameter), and internal vascularity (no internal flow, rare internal flow and rich internal flow). All US images were analyzed by two blind radiologists (Xu JM, who had 13 years’ experience with thyroid US and 5 years’ experience with thyroid elastography; Liu BJ, who had 3 years’ experience with thyroid US and 3 years’ experience with thyroid elastography) and disagreement was solved by consensus.

**Figure 4:**
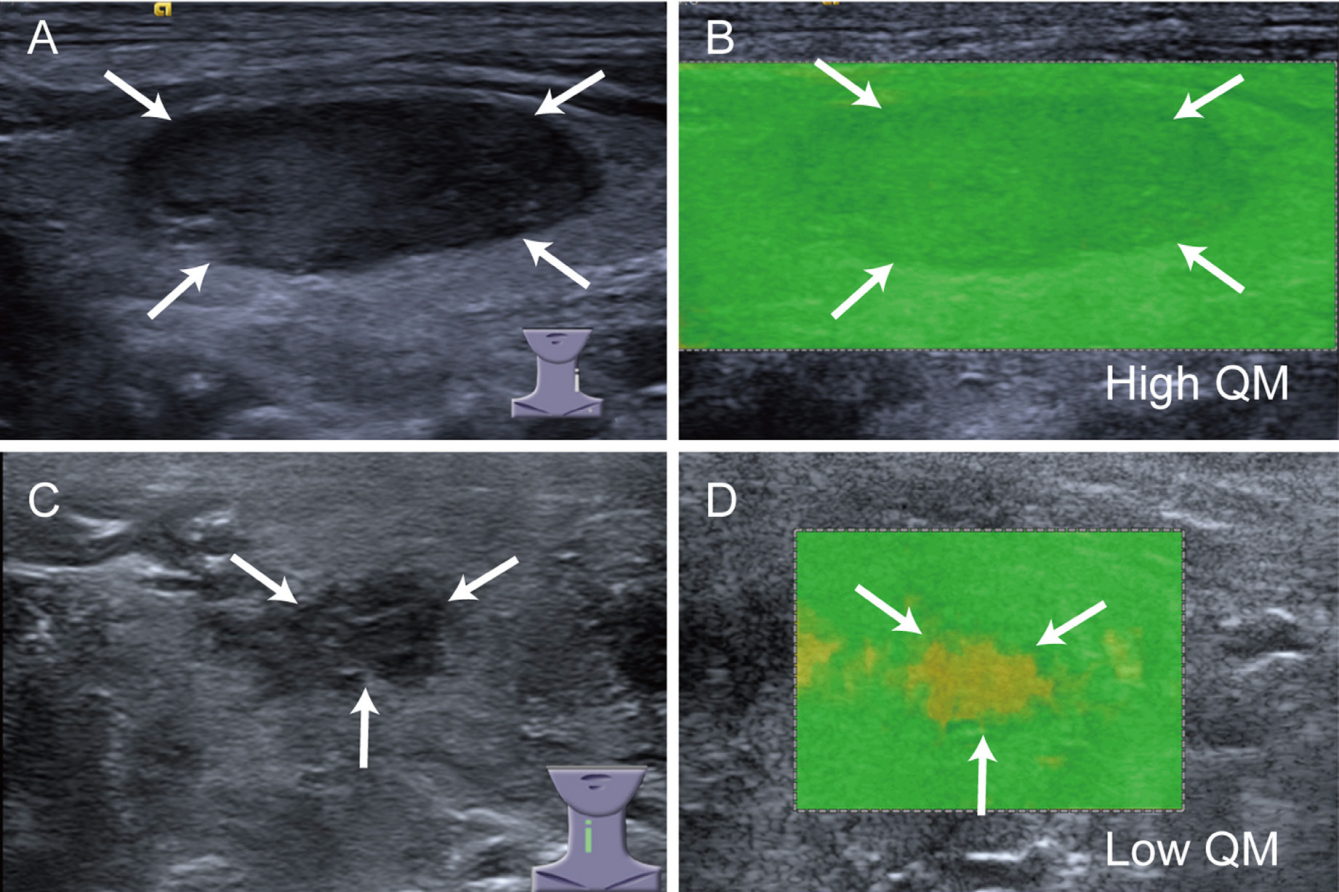
SW-quality images of high QM and low QM Low QM is defined that internal yellow portions are more than 20 percent of the nodule (arrows), otherwise high QM is determined.

When the quality image was nearly green or green portions accounted for more than 80 percent, it was classified as high quality; otherwise, low quality was defined (Figure 4). According to our experience, if the nodules have 80% green portions on quality image, it does not affect the following ROI placement, thus we defined low quality as above mentioned. For the nodules with < 25% cystic portions, the cystic portions did not impact the quality analysis and only the solid portion was focused since SW does not propagate in fluid areas. All SW quality images were analyzed by the same two blind radiologists using another setting after training of 30 cases. Mean SWS of each included nodule was calculated and used for analysis.

### Fine need aspiration (FNA)

Solid nodules with diameter more than 10mm and solid-cystic mixed nodules more than 20mm were recommended for FNA [27]. On the other side, < 10 mm nodules which had one of the following markedly suspicious US features including marked hypoechogenicity, taller than wide shape, multiple microcalcifications and extrathyroidal extension were recommended for FNA. Only the patients who had normal serial leukocyte, thrombocyte, coagulation function and no serious cardiopulmonary diseases could receive FNA. All FNA procedures were guided by US and performed by one of three experienced operators who had more than 3 years’ experience with FNA using a 22-gauge, 15 cm long needle after local anesthesia. The obtained sample in the needle was mounted on a glass slide immediately and put in 75% alcohol solution for fixation. No less than 3 glass slides were obtained to make sure that sample was enough.

FNA cytological results are classified as 6 categories according to Bethesda system [1], they were: (I) non-diagnostic; (II) benign; (III) atypia of undetermined significance/follicular lesion of undetermined significance; (IV) follicular neoplasm/suspicious for follicular neoplasm; (V) suspicious for malignancy, and (VI) malignant. Bethesda II results were recommended to follow up while Bethesda V and VI results to surgery. For non-diagnostic cytological results, repeat US-guided FNA was recommended. For Bethesda III and IV results, diagnostic surgery or repeat FNA was recommended.

### Statistical analysis

Statistical analysis was performed using SPSS software (version 20.0; SPSS, Chicago, III). A two-tailed *P* value < 0.05 indicated statistically significant difference. Continuous variables were expressed as mean ± standard deviation (SD) when they fitted normal distribution. Continuous variables were analyzed using independent t test, while categorical variables were analyzed using nonparametric Mann–Whitney U test. Binary counted variables were analyzed using χ*^2^* test. ROC curves of SWS and combination of SWS and QM were performed and AUCs were obtained, the cut-off points were selected when Youden index (sensitivity+specificity-1) reached the maximum values. Based on these cut-off values, sensitivity, specificity, PPV, NPV and accuracy were calculated. Comparison of sensitivity, specificity and accuracy between SWS, QM and SWS+QM were performed using Mc-Nemar test while PPV and NPV was compared by χ*^2^* test. Comparison of AUCs was performed by using Z test described by Hanley [28]. Multivariate logistic regression analysis was performed to assess 12 possible associated factors for low QM prediction. The diagnostic value of QM and SWS+QM by two radiologists was also calculated and compared. Subgroup analysis of diameter (< 10 mm group and ≥ 10 mm group) and depth (≤ 15 mm group and > 15 mm group) were performed.

## CONCLUSIONS

QM for thyroid nodule is associated with nodule depth, malignancy, and SWS. Low QM is more often happened when deeper thyroid nodule, thyroid malignancy, and high SWS is encountered. QM significantly improves the diagnostic specificity, whereas the sensitivity decreases. Furthermore, adding QM does not improve the diagnostic performance in comparison with SWS alone. QM should be used as a reference to place SW ROI for SWS measurement to guarantee reliable SWS measurement, rather than for diagnosis of thyroid nodules.
